# Performance of CHA_2_DS_2_-VASc and HAS-BLED in predicting stroke and bleeding in atrial fibrillation and cancer

**DOI:** 10.1093/ehjopen/oeae053

**Published:** 2024-06-26

**Authors:** Alyaa M Ajabnoor, Salwa S Zghebi, Rosa Parisi, Darren M Ashcroft, Corinne Faivre-Finn, Mamas A Mamas, Evangelos Kontopantelis

**Affiliations:** Department of Pharmacy Practice, Faculty of Pharmacy, King Abdulaziz University, P.O. Box 80324, Jeddah 21589, Saudi Arabia; Division of Informatics, Imaging and Data Sciences, School of Health Sciences, Faculty of Biology, Medicine and Health, Manchester Academic Health Science Centre (MAHSC), University of Manchester, Manchester M13 9PL, UK; Division of Population Health, Health Services Research and Primary Care, School of Health Sciences, Faculty of Biology, Medicine and Health, Manchester Academic Health Science Centre (MAHSC), University of Manchester, Manchester M13 9PL, UK; Division of Informatics, Imaging and Data Sciences, School of Health Sciences, Faculty of Biology, Medicine and Health, Manchester Academic Health Science Centre (MAHSC), University of Manchester, Manchester M13 9PL, UK; Division of Pharmacy and Optometry, Centre for Pharmacoepidemiology and Drug Safety, School of Health Sciences, Faculty of Biology, Medicine and Health, University of Manchester, Manchester M13 9PL, UK; National Institute for Health and Care Research (NIHR) Greater Manchester Patient Safety Research Collaboration (PSRC), University of Manchester, Manchester M13 9PL, UK; The Christie NHS Foundation Trust and The University of Manchester, Manchester, UK; Keele Cardiovascular Research Group, Centre for Prognosis Research, Institute for Primary Care and Health Sciences, Keele University, Keele, UK; National Institute for Health and Care Research (NIHR) Birmingham Biomedical Research Center, UK; Division of Informatics, Imaging and Data Sciences, School of Health Sciences, Faculty of Biology, Medicine and Health, Manchester Academic Health Science Centre (MAHSC), University of Manchester, Manchester M13 9PL, UK

**Keywords:** Atrial fibrillation, Cancer, Stroke, Bleeding, Risk assessment score, Oral anticoagulant

## Abstract

**Aims:**

To compare the predictive performance of CHA_2_DS_2_-VASc and HAS-BLED scores in atrial fibrillation (AF) patients with and without cancer.

**Methods and results:**

Using data from the Clinical Practice Research Datalink in England, we performed a retrospective cohort study of patients with new diagnoses of AF from 2009 to 2019. Cancer was defined as history of breast, prostate, colorectal, lung, or haematological cancer. We calculated the CHA_2_DS_2_-VASc and HAS-BLED scores for the 1-year risk of stroke and major bleeding events. Scores performance was estimated by discrimination [area under the receiver operating characteristic curve (AUC)] and calibration plots. Of 141 796 patients with AF, 10.3% had cancer. The CHA_2_DS_2_-VASc score had good to modest discrimination in prostate cancer AUC = 0.74 (95% confidence interval: 0.71, 0.77), haematological cancer AUC = 0.71 (0.66, 0.76), colorectal cancer AUC = 0.70 (0.66, 0.75), breast cancer AUC = 0.70 (0.66, 0.74), and lung cancer AUC = 0.69 (0.60, 0.79), compared with no-cancer AUC = 0.73 (0.72, 0.74). HAS-BLED discrimination was poor in prostate cancer AUC = 0.58 (0.55, 0.61), haematological cancer AUC = 0.59 (0.55, 0.64), colorectal cancer AUC = 0.57 (0.53, 0.61), breast cancer AUC = 0.56 (0.52, 0.61), and lung cancer AUC = 0.59 (0.51, 0.67), compared with no-cancer AUC = 0.61 (0.60, 0.62). Both the CHA_2_DS_2_-VASc score and HAS-BLED score were well calibrated across all study cohorts.

**Conclusion:**

Amongst certain cancer cohorts in the AF population, CHA_2_DS_2_-VASc performs similarly in predicting stroke to AF patients without cancer. Our findings highlight the importance of cancer diagnosis during the development of risk scores and opportunities to optimize the HAS-BLED risk score to better serve cancer patients with AF.

## Graphical Abstract

**Graphical Abstract:**
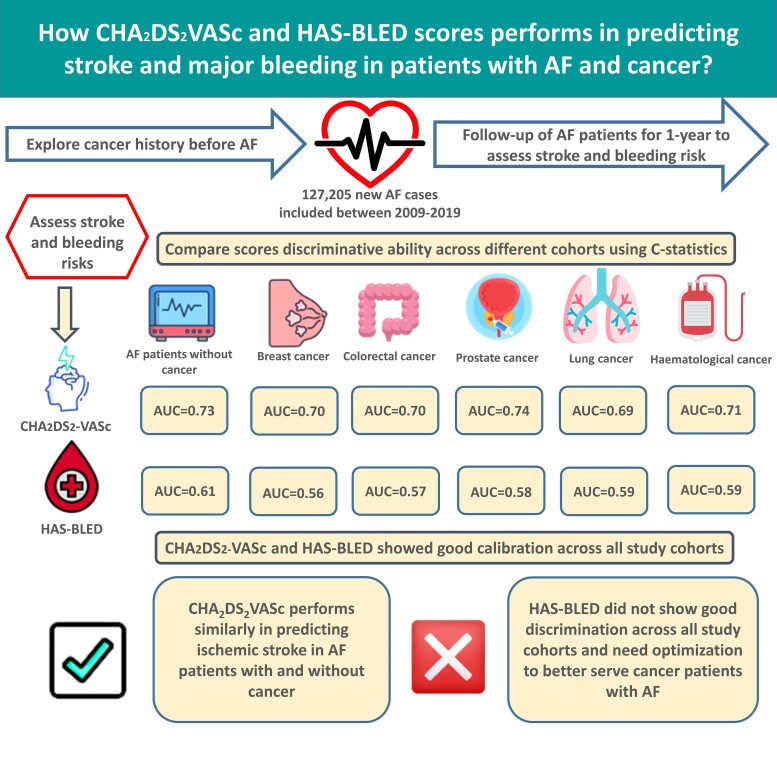

## Background

Atrial fibrillation (AF) is more prevalent in the cancer population than in the general population, due to several pathophysiological mechanisms that have been found to induce AF in cancer patients.^1,2^ Of which, cancer-related systemic inflammation can contribute to atrial remodelling,^1,3–5^ electrolyte and metabolic abnormalities, fluid imbalance (e.g. during chemotherapy) and infections.^3^ CHA_2_DS_2_-VASc score is commonly utilised to predict thrombo-embolic risk in patients with AF and its utilization is recommended by guidelines to guide the prescription of oral anticoagulants (OAC).^6^ In individuals with cancer, the risk of stroke is increased in most cancer types, particularly in the period after diagnosis.^7,8^ This could be secondary to the hypercoagulable state associated with cancer and the prothrombotic effect increased by some types of chemotherapies that could also increase the risk of bleeding.^9^ Cancer history is not considered in the CHA_2_DS_2_-VASc score, and so its performance in the cancer population may be suboptimal, particularly since the score does not consider risk factors specific to cancer patients. Similarly, the HAS-BLED score was developed to predict bleeding risk in AF patients and includes both modifiable and non-modifiable risk factors. Guidelines recommend that HAS-BLED should be used to identify patients at high risk of bleeding.^6^ In patients with cancer, bleeding risk is much higher than in the general population, due to treatment- or disease-related thrombocytopenia, and coagulopathies, respectively. HAS-BLED also does not take into account malignancy as a risk factor for bleeding. In clinical practice, the assessment of bleeding risk in AF patients with cancer takes place on a case-by-case basis, focusing on cancer type, cancer stage, treatment type, and physical fitness.

The association between cancer and ischaemic or bleeding outcomes in patients with AF is unclear, and the incidence of thrombo-embolic events and bleeding reported for the cancer population in recent clinical trials of non-vitamin K antagonist oral anticoagulants showed varying results.^10–13^ Moreover, there is limited information on the performance of the currently validated risk scores (i.e. CHA_2_DS_2_-VASc and HAS-BLED) in different types of cancer.

Using a population-based electronic health record dataset linking primary and secondary care data and mortality records in England, we evaluated the performance of the CHA_2_DS_2_-VASc score and HAS-BLED score in patients with AF and five types of cancer. We investigated their ability to predict ischaemic stroke and major bleeding events in different types of cancer.

## Methods

### Study design

This was a population-based retrospective cohort study using data from the Clinical Practice Research Datalink (CPRD) GOLD and Aurum databases, with data linkage to Hospital Episode Statistics (HES) for admitted patients, Office of National Statistics (ONS) death registration,^14,15^ and the 2015 Index of Multiple Deprivation (IMD).^16^ The CPRD is a longitudinal primary care database of anonymized general practitioner medical records in the UK and is broadly representative of the UK population. CPRD GOLD represents around 7% of the UK population, and CPRD Aurum represents around 13%, respectively, and contains consultation records, patient demographic information, diagnoses, drug prescriptions, and referrals to secondary care.^14,15^ We only included data from English practices consented to data linkages to IMD, HES, and ONS. All relevant clinical factors were assessed and explored at baseline using Read codes alone in CPRD GOLD or using both SNOMED/EMIS and Read codes in CPRD Aurum.

### Study population

We included new diagnoses of AF recorded between 1 January 2009 and 31 December 2019. Subjects were included if they were adults aged ≥18 years and registered with an English general practice contributing data to the CPRD for ≥1 year before AF diagnosis, and with no prior valvular pathology coded in the health records. We applied exclusion criteria within a lookback period of 12 months before AF diagnosis: records of irregular heartbeats or cardioversion, records of atrial flutter alone with no mention of AF, previous use of quinidine, sotalol, amiodarone, flecainide, or propafenone, and previous use of oral or parenteral anticoagulants *>*14 days before AF diagnosis. We focused on the most common cancer types diagnosed in England^17^; breast, prostate, colorectal, and lung cancer, which are also associated with cardiovascular disease.^18^ We have also included patients with haematological malignancy because it is known to increase bleeding risk.^19^ Only patients with incident AF and a diagnosis of these five types of cancer at any time point before AF were included in the cancer group, and patients with other types of cancer were excluded from the analysis. All patients were followed for 12 months from the start of their index date until the earliest occurrence of the outcomes of interest (i.e. stroke or bleeding), end of 1-year observation period, cancer diagnosis (in case of the comparison population), or death. This duration of 1 year of follow-up was chosen due to its clinical relevance while allowing for sufficient follow-up time for the estimation of risk.

### Covariates and calculation of risk scores

The nine-point CHA_2_DS_2_-VASc score and HAS-BLED score were calculated according to the original work by Lip *et al.*^20^ and Pisters *et al.*^21^ (see Supplementary material online, *Table S1*). The definition of uncontrolled hypertension [systolic blood pressure (SBP) > 160 mm Hg] was supplemented with data on prescribing of three concurrent antihypertensive drugs from different classes if prescribed within 90 days before the index date.^22^ SBP was assessed during 90 days before the index date. Labile international normalized ration (INR) was unavailable because data on INR are inconsistently recorded in CPRD, therefore labile INR was scored 0 point and a modified HAS-BLED of eight points was used instead. Diagnostic codes were independently reviewed by an expert cardiologist (M.A.M.), and medication lists were reviewed by the 1st author who is a pharmacist (A.M.A.). Exposure to OAC was assessed by considering the 1st continuous treatment episode during the 1 year of follow-up after AF diagnosis.^23^

### Definition of outcomes

The main outcomes of the study are; 1-year risk of ischaemic stroke defined as the first occurrence of the event in either CPRD, HES, and ONS records after AF and within a year, and/or major bleeding defined as a hospital record in HES data after the index date of AF diagnosis which could be either (i) bleeding occurring at a critical site (i.e. intracranial, intraspinal, intraocular, pericardial, intra-articular, intramuscular, and retroperitoneal), (ii) bleeding that led to hospitalization, or (iii) fatal bleeding (as a cause of death identified in ONS records).^24^ All outcomes were identified using CPRD codes for primary care events, ICD-10 codes for HES, or ONS records. The codes used to produce the data and the scores for this study can be found at https://github.com/ammajabnour/AF-project.

### Statistical analysis

Baseline characteristics are presented as frequencies (%) for categorical data, medians, and interquartile ranges (IQRs) for non-normally distributed continuous data or means and standard deviation for normally distributed continuous data. In the main analysis of the models (i.e. CHA_2_DS_2_-VASc score and HAS-BLED score), we calculated the 1-year risk of either stroke or bleeding using clinical records from CPRD, HES, and ONS records. The cumulative incidence of stroke and bleeding events over the follow-up period was plotted using Kaplan–Meier curves and risk tables were calculated to provide number of followed and censored patients. Missing body mass index (BMI) values at baseline were imputed by an interpolation algorithm that has been used in previous studies using the CPRD.^25^ A previously used algorithm was also used to manage smoking status inconsistencies at baseline.^26^ As for the performance of the scores, the scores were modelled as categorical variables with aggregation (1, 2, 3, 4, 5, > 6). We first assessed the overall discrimination score by determining the area under the receiver operating characteristic curve (AUC), before calculating the AUC separately in each cancer group. Labelling systems for AUC are usually arbitrary,^27^ and for the purpose of this study we labelled an AUC of ≥0.70 as good, ≥ 0.60 as modest, and <0.60 as poor. Following that, we assessed calibration in each patient group, by running a regression model to predict the 1-year risk of ischaemic stroke or major bleeding, before plotting it using the pmcalplot command in Stata. We examined several properties from the calibration curve, including (i) the calibration in the large (CITL); (ii) the calibration slope (>1 overestimate risk, whilst a slope of <1 underestimate risk); and (iii) the expected: observed (E:O) ratio, where strong calibration would result in a ratio of 1 with expected and observed rates being similar.^28^ We also calculated and compared diagnostic utility sensitivity, specificity, positive predictive values (PPV), and negative predictive values (NPV) for the original CHA_2_DS_2_-VASc score and HAS-BLED score and when cancer was added as a variable. Furthermore, we examined both the net reclassification index (NRI) and the integrated discrimination index by adding cancer as a covariate to CHA_2_DS_2_-VASc score and HAS-BLED score.

We conducted several sensitivity analyses to test the robustness of the produced results, first we estimated the AUC of the two scores if the scores were calculated at baseline using only primary care data. Second, we estimated the AUC of the two scores when modelled as continuous variables and not as factors. Third, we estimated the AUC of the two scores if they were modelled as categorical variables with aggregation and when cancer was defined as cancer diagnosed within 2 years before AF. Finally, we have estimated the AUC for the two scores and included only patients who did not take OAC during the 1-year period of the study.

All statistical analyses were performed using Stata 16 (Stata Corp., College Station, TX, USA), they were two-tailed with an alpha of 5% used throughout. This study is reported in line of the Transparent reporting of a multivariable prediction model for individual prognosis or diagnosis (TRIPOD) statement.

## Results

### Baseline characteristics

**Figure 1:**
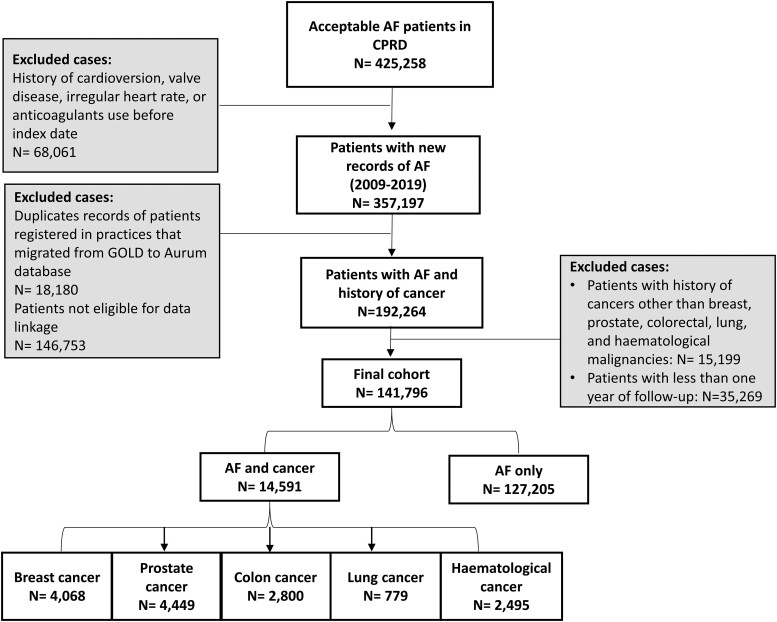
Cohort selection and number of included and excluded patients.

Applying the inclusion/exclusion criteria produced a study cohort consisting of 141 796 patients with AF (*Figure 1*). Of these, 14 591 patients had history of cancer [breast cancer *n* = 4068 (27.9%), prostate cancer *n* = 4449 (30.4%), colorectal cancer *n* = 2800 (19.2%), haematological cancer *n* = 2495 (17%), and lung cancer *n* = 779 (5.3%)]. *Table 1* describes the differences in baseline characteristics across cancer and patients without cancer. Cancer patients were older (median age 79 years, IQR = 72–84) compared with patients without cancer 73 years (66–82). The majority of patients (>90%) across all cohorts was from white ethnicity. Compared with other cancer types and patients without cancer, patients with lung cancer had the highest proportion of patients from the most deprived quintile (IMD 5) (22.8%) compared with other study cohorts (11.5–14.5%). In addition, lung cancer patients had the lowest BMI and were more likely to be smokers, and heavy alcohol drinkers (*Table 1*). The proportion of patients at high stroke risk or at high bleeding risk is overall higher in cancer patients (78–93.2% for stroke risk and 38.7–50.8% for bleeding risk) compared with 79.3% and 36.8% in patients without cancer. This is related to the higher prevalence of chronic conditions across cancer patients compared with patients without cancer. More noticeably for hypertension, diabetes, chronic kidney disease, heart failure, and anaemia that were more common in cancer patients.

**Table 1:** Clinical characteristics of atrial fibrillation patients stratified by cancer status

Characteristic	AF (without cancer)	AF patients with cancer				
		Breast cancer	Prostate cancer	Colorectal cancer	Haematological cancer	Lung cancer
	*n* = 127 205	*n* = 4068	*n* = 4449	*n* = 2800	*n* = 2495	*n* = 779
Median age (IQR)	73 (66–82)	77 (72–84)	78 (73–85)	78 (74–84)	76 (70–83)	75 (70–81)
Males, *n* (%)	69 280 (54.5)	42 (1)	4448 (100)	1603 (57.3)	1452 (58.2)	422 (54.2)
Median BMI (IQR)	29 (25–28)	28 (24–32)	28 (24–31)	28 (24–31)	28 (24–31)	24 (23–30)
Current smoker, *n* (%)	26 448 (21)	702 (17.3)	773 (17.4)	466 (16.6)	491 (19.7)	312 (40.1)
Heavy alcohol consumption, *n* (%)	13 815 (10.9)	272 (6.7)	509 (11.4)	277 (9.9)	214 (8.6)	101 (13)
Ethnicity, *n* (%)						
White	116 554 (91.6)	3900 (95.9)	4233 (95.2)	2702 (96.5)	2384 (95.5)	752 (96.5)
Black	1096 (0.9)	31 (0.8)	84 (1.9)	24 (0.8)	19 (0.8)	<5 (0.4)
Asian	1869 (1.4)	38 (0.9)	46 (1)	17 (0.6)	35 (1.4)	9 (1.2)
Others	1121 (0.9)	30 (0.7)	22 (0.5)	13 (0.5)	20 (0.8)	6 (0.8)
Missing	6566 (5.2)	69 (1.7)	63 (1.4)	44 (1.6)	37 (1.5)	9 (1.1)
Socioeconomic status, *n* (%)						
IMD 1 (least deprived)	31 666 (24.9)	1050 (25.8)	1281 (28.8)	725 (25.9)	662 (26.5)	160 (20.5)
IMD 2	28 570 (22.5)	941 (23.1)	1053 (23.7)	620 (22.1)	563 (22.6)	149 (19.1)
IMD 3	26 317 (20.6)	889 (21.9)	918 (20.6)	577 (20.6)	525 (21)	142 (18.2)
IMD 4	22 264 (17.5)	646 (15.9)	684 (15.4)	492 (17.6)	421 (16.9)	151 (19.4)
IMD 5 (most deprived)	18 389 (14.5)	542 (13.3)	512 (11.5)	386 (13.8)	324 (13)	177 (22.8)
Chronic conditions, *n* (%)						
Hypertension	79 183 (62.3)	2764 (67.9)	3014 (67.8)	1946 (69.5)	1596 (64)	499 (64.1)
Diabetes mellitus	25 915 (20.4)	814 (20)	960 (21.6)	670 (23.9)	585 (23.5)	166 (21.3)
Chronic kidney disease	26 012 (20.5)	1078 (26.5)	1127 (25.3)	762 (27.2)	736 (29.5)	173 (22.2)
Liver failure	1055 (0.83)	31 (0.76)	21 (0.47)	25 (0.89)	25 (1)	8 (1.03)
Heart failure	11 315 (8.9)	366 (9)	519 (11.7)	257 (9.2)	317 (12.7)	80 (10.3)
Ischaemic heart disease	36 082 (28.4)	971 (23.9)	1589 (35.7)	877 (31.3)	787 (31.5)	228 (29.3)
History of stroke/TIA	18 037 (14.2)	594 (14.6)	747 (16.8)	459 (16.4)	388 (15.6)	95 (12.2)
History of major bleeding	21 216 (16.7)	587 (14.4)	1151 (25.6)	599 (21.4)	513 (20.6)	137 (17.6)
Anaemia	18 077 (14.2)	715 (17.6)	796 (17.9)	804 (28.7)	655 (26.3)	144 (18.5)
Dementia	3168 (2.5)	148 (3.6)	121 (2.7)	81 (2.9)	52 (2.1)	13 (1.7)
Peripheral vascular disease	6170 (4.9)	171 (4.2)	292 (6.6)	189 (6.8)	149 (6)	78 (10)
Pulmonary embolism	1695 (1.3)	98 (2.4)	74 (1.7)	71 (2.5)	63 (2.5)	15 (1.9)
Deep venous thrombosis	4187 (3.3)	220 (5.4)	189 (4.3)	119 (4.3)	130 (5.2)	24 (3.1)
Peptic ulcer	6366 (5)	180 (4.4)	324 (7.3)	216 (7.7)	168 (6.7)	70 (9)
Low^a^ stroke risk	11 441 (9)	125 (3.1)	40 (0.9)	57 (2)	70 (2.8)	26 (3.3)
Intermediate^a^ stroke risk	14 834 (11.7)	350 (8.6)	262 (5.9)	165 (5.9)	227 (9.1)	75 (9.6)
High^a^ stroke risk	100 931 (79.3)	3593 (88.3)	4146 (93.2)	2578 (92.1)	2198 (88.1)	678 (87)
Median CHA_2_DS_2_VASc score (IQR)	4 (2–5)	4 (3–5)	4 (3–5)	4 (3–5)	4 (3–5)	4 (3–5)
Low^b^ bleeding risk	43 581 (34.3)	1154 (28.4)	853 (19.2)	548 (19.6)	598 (24)	205 (26.3)
Intermediate^b^ bleeding risk	36 843 (29)	1340 (32.9)	1346 (30.3)	829 (29.6)	736 (29.5)	247 (31.7)
High^b^ bleeding risk	46 782 (36.8)	1574 (38.7)	2249 (50.6)	1423 (50.8)	1161 (46.5)	327 (42)
Median HAS-BLED score (IQR)	2 (1–3)	2 (1–3)	3 (2–3)	3 (2–4)	2 (2–3)	2 (1–3)
Time from cancer to AF diagnosis, *n* (%)						
Within 6 months	NA^a^	660 (16.2)	1744 (39.2)	697 (24.8)	1155 (46.3)	464 (59.6)
Within 2 years		561 (13.8)	1038 (23.3)	436 (15.6)	475 (19)	115 (14.7)
Within 5 years		786 (19.3)	822 (18.5)	550 (19.6)	383 (15.4)	82 (10.5)
Over 5 years		2061 (50.7)	844 (19)	1117 (40)	482 (19.3)	118 (15.2)

AF, atrial fibrillation; BMI, body mass index; IMD, index of multiple deprivation; TIA, transient ischaemic attack.

^a^Stroke risk: low, CHA_2_DS_2_-VASc score is equal to 0 in males, or 1 in females; intermediate, CHA_2_DS_2_-VASc score equal 1 in males, or 2 in females; high, CHA_2_DS_2_-VASc score ≥2 in males, or ≥3 in females.

^b^Bleeding risk: low, HAS-BLED is 0 or 1; intermediate, HAS-BLED is 2; high, HAS-BLED is ≥3.

### Incidence rates of stroke and bleeding events

The crude incidence rate and 95% confidence interval (CI) of ischaemic stroke during the 1-year follow-up was 5.2 (5.0–5.38) per 100 person-years at risk (PYR) for AF patients without cancer, 6.1 (5.4–6.9) for breast cancer, 5.9 (5.1–6.9) for colorectal cancer, 5.6 (4.9–6.4) for prostate cancer, 5.3 (4.5–6.3) for haematological cancer, and 4.9 (3.5–6.7) for lung cancer (*Table 2*). The incidence rate was also demonstrated in the Kaplan–Meier curve and risk table (see Supplementary material online, *Figure S1*) that shows the reduction of number of patients followed in each study cohort during the follow-up period. In the group without cancer, 68.7% of patients with recommendation for OAC were on anticoagulants during the 1-year observation window (*Table 2*). Whereas in cancer patients, 68.8% with breast cancer received OAC, prostate cancer 68.6%, colorectal cancer 63.5%, haematological cancer 62.3%, and lung cancer 56.2%. Supplementary material online, *Table S2* provides data on the rate of stroke events per score value for AF patients, with and without cancer, who did not start anticoagulation therapy. It shows that in both cohorts (AF with and without cancer), the CHA_2_DS_2_-VASc score performs very well in identifying patients at low risk (<1% stroke event in 1 year), those with a CHA_2_DS_2_-VASc score of 0 or 1. Moreover, in the cancer cohort, the CHA_2_DS_2_-VASc score allowed identification of a truly low-risk population those with CHA_2_DS_2_-VASc = 0 and in whom no stroke events were recorded. The crude incidence rate of major bleeding events and 95% CI was 4.5 (4.4–4.6) per 100 person-years for the cohort without cancer, 8.5 (7.7–9.5) for prostate cancer, 6.6 (5.0–8.7) for lung cancer, 6.6 (5.7–7.6) for colorectal cancer, 6.4 (5.5–7.5) for haematological cancer, and 3.8 (3.3; 4.5) for breast cancer (*Table 2* and Supplementary material online, *Figure S2*).

**Table 2:** Incidence rates and 95% CI of ischaemic stroke and major bleeding events per 100 person-years at risk of follow-up during the 1-year observation window

Characteristic	AF (without cancer)	AF patients with cancer				
		Breast cancer	Prostate cancer	Colorectal cancer	Haematological cancer	Lung cancer
	*n* = 127 205	*n* = 4068	*n* = 4449	*n* = 2800	*n* = 2495	*n* = 779
Patients prescribed OAC^a^	82 047 (64.5)	2471 (63.5)	2842 (68.5)	1637 (63.5)	1369 (62.3)	381 (56.2)
Incidence rate of ischaemic stroke per 100 PYR	5.2 (5.0–5.3)	6.1 (5.4–6.9)	5.6 (4.9–6.4)	5.9 (5.1–6.9)	5.3 (4.5–6.3)	4.9 (3.5–6.7)
Time to first stroke event [median days, IQR]	65 (6–204)	77 (7–209)	84 (13–225)	59 (6–199)	104 (7–230)	146 (32–266)
Incidence rate of major bleeding events per 100 PYR	4.5 (4.4–4.6)	3.8 (3.3–4.5)	8.5 (7.7–9.5)	6.6 (5.7–7.6)	6.4 (5.5–7.5)	6.6 (5.0–8.7)
Time to first bleeding event (median days)	139 (46–249)	165 (68–260)	166 (73–264)	125 (56–225)	132 (58–224)	189 (99–286)

^a^Estimates for patients with the recommendation of OAC based on stroke risk (CHA_2_DS_2_-VASc ≥2 in males, and ≥3 in females).

### Discrimination and calibration

**Figure 2:**
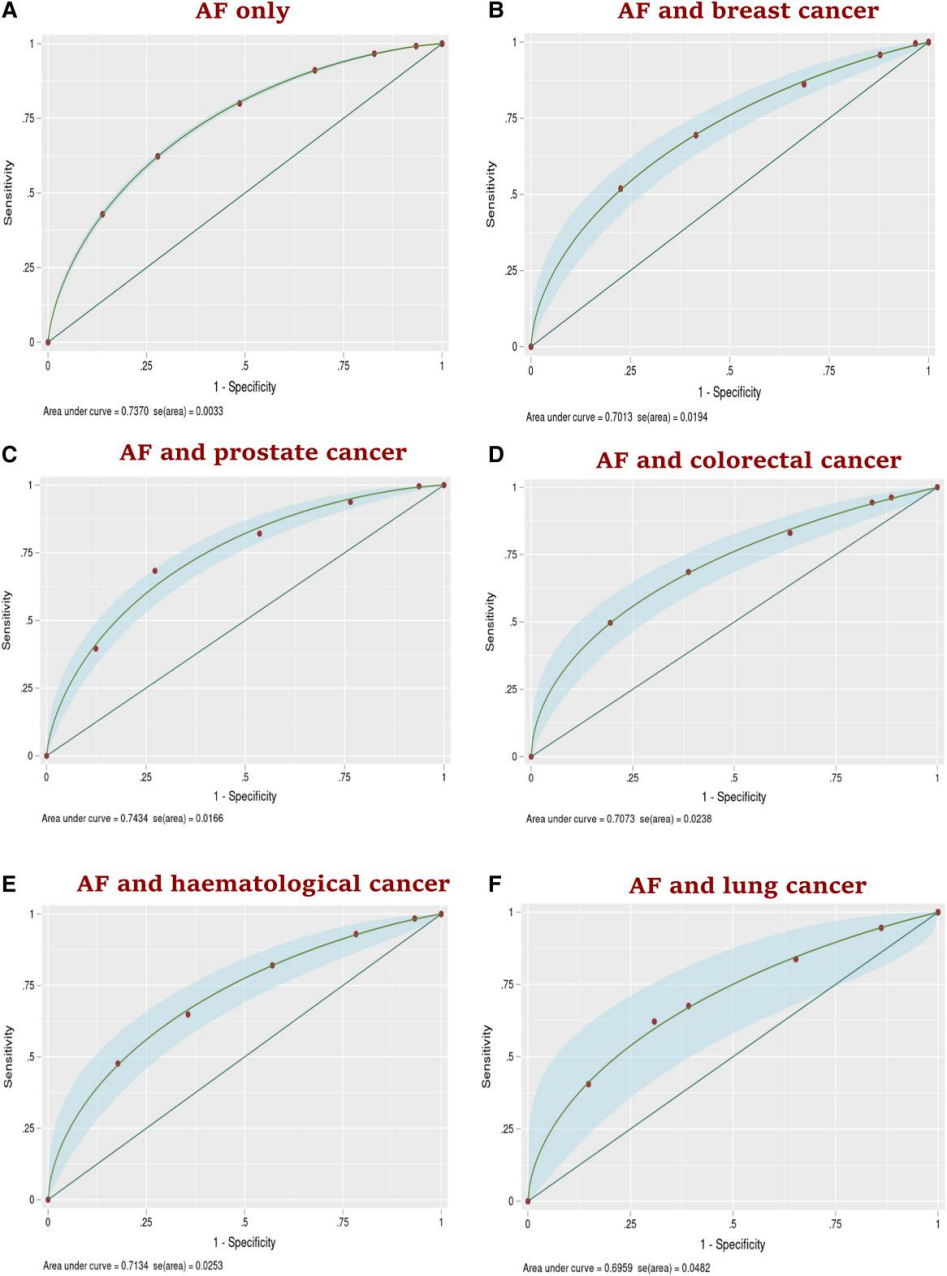
Discrimination plots (*AitalicF*) of the CHA_2_DS_2_-VASc risk model for 1-year risk of stroke in atrial fibrillation patients with and without cancer.

**Figure 3:**
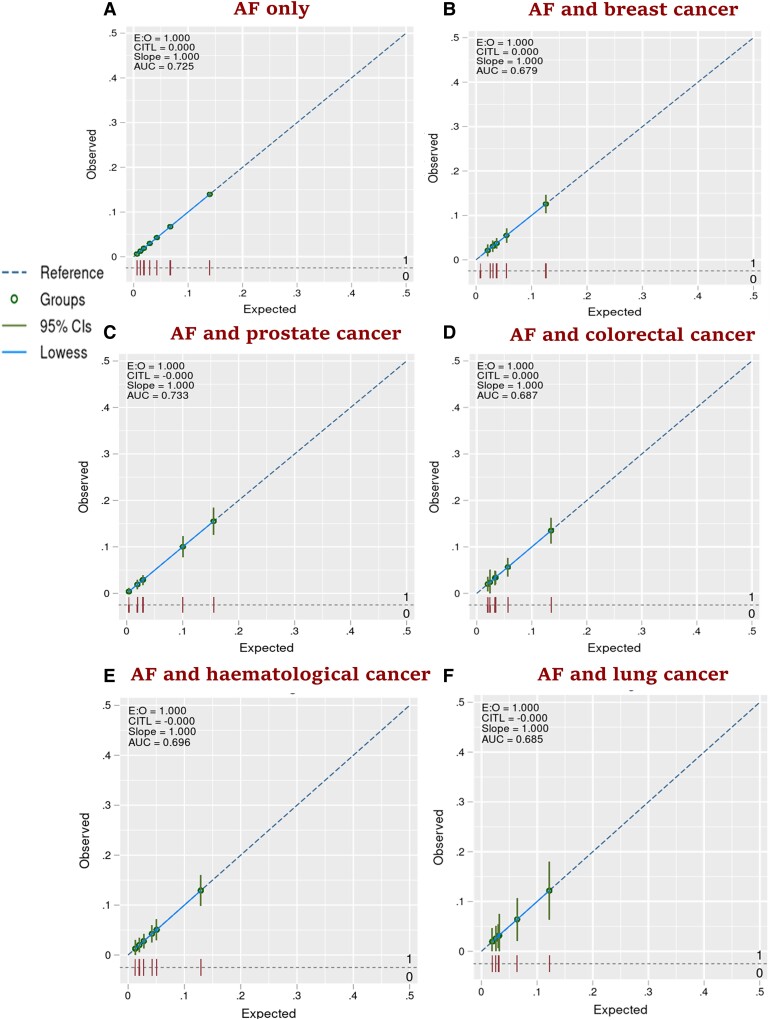
Calibration plots (*AitalicF*) of the CHA_2_DS_2_-VASc risk model for 1-year risk of stroke in atrial fibrillation patients with and without cancer.

**Figure 4:**
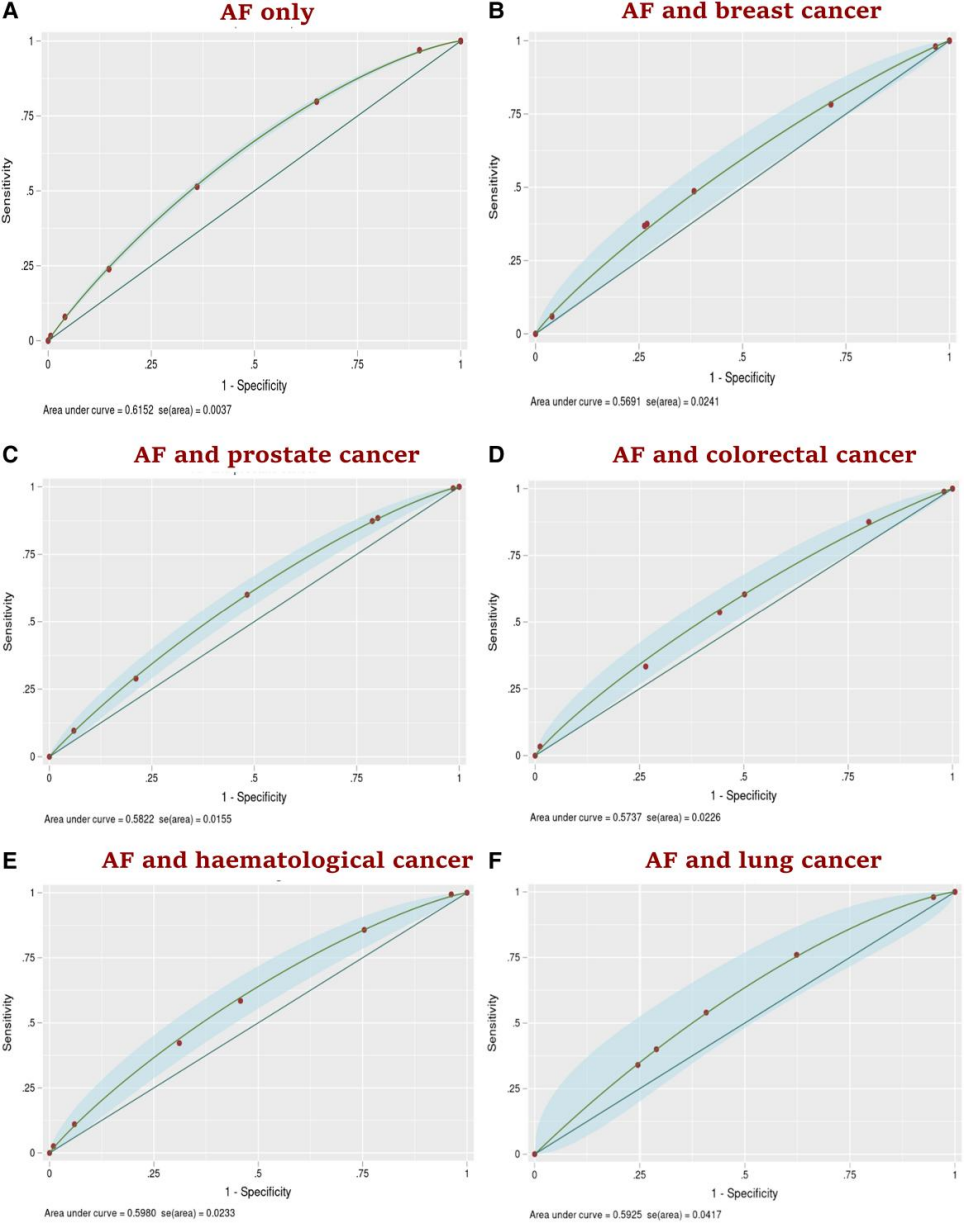
Discrimination plots (*AitalicF*) of the HAS-BLED risk model for 1-year risk of bleeding in atrial fibrillation patients with and without cancer.

**Figure 5:**
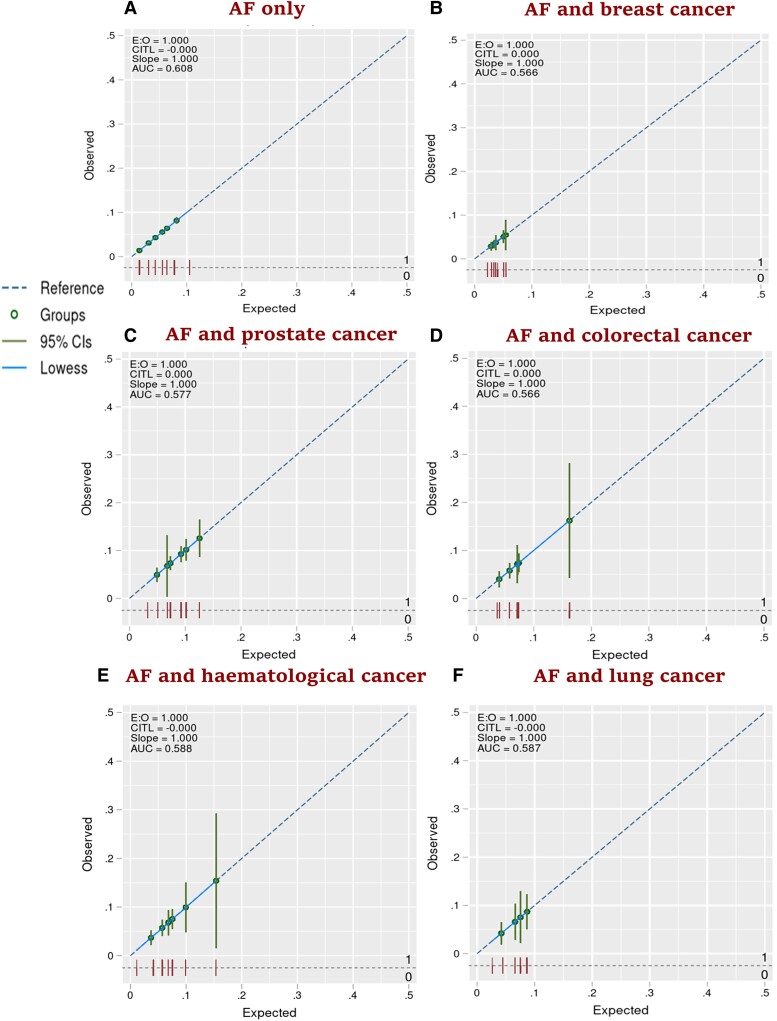
Calibration plots (*AitalicF*) of the HAS-BLED risk model for 1-year risk of major bleeding in atrial fibrillation patients with and without cancer.

For the cohort without cancer, the CHA_2_DS_2_-VASc score had good discrimination: AUC of 0.73 (95% CI: 0.72, 0.74) (*Figure 2*). It also had good discrimination in prostate cancer (AUC = 0.74; 95% CI: 0.71, 0.77), haematological cancer (AUC = 0.71; 95% CI: 0.66, 0.76), colorectal cancer (AUC = 0.70; 95% CI: 0.66, 0.75), and breast cancer cohorts (AUC = 0.70; 95% CI: 0.66, 0.74). However, the CHA_2_DS_2_-VASc score showed modest discrimination in and lung cancer cohort (AUC = 0.69; 95% CI: 0.60, 0.79). The calibration plots suggest that the CHA_2_DS_2_-VASc score was well calibrated in the cohort without cancer, and all five cancer groups, with an E:O ratio of 1.000, CITL value of 0.000, and slope of 1.00 (*Figure 3*). For the cohort without cancer, the HAS-BLED score had modest discrimination with an AUC of 0.61 (95% CI: 0.60, 0.62) (*Figure 4*). HAS-BLED discrimination was poor across all cancer cohorts: haematological cancer (AUC = 0.59; 95% CI: 0.55, 0.64), lung cancer (AUC = 0.59; 95% CI: 0.51, 0.67), prostate cancer (AUC = 0.58; 95% CI: 0.55, 0.61), colorectal cancer (AUC = 0.57; 95% CI: 0.53, 0.61), and breast cancer (AUC = 0.56; 95% CI: 0.52, 0.61). The calibration plots for HAS-BLED (*Figure 5*) suggest that the score was well calibrated in the group without cancer and in all five cancer groups, with an E:O ratio of 1.000, and slope of 1.00.

### Sensitivity analysis

When the CHA_2_DS_2_-VASc score was modelled as continuous variable, the score discrimination was similar to what was observed in the main analysis (see Supplementary material online, *Table S3*). The only potential exception was the breast cancer cohort, with an AUC of 0.68 (95% CI: 0.65, 0.72), compared with an AUC of 0.70 (95% CI: 0.66, 0.74) in the main analysis. Similar to the main analysis, the CHA_2_DS_2_-VASc score was well calibrated across all study cohorts. Using primary care data alone to build the CHA_2_DS_2_-VASc score resulted in a score with 5% lower discrimination, on average across all study cohorts, compared with the main analysis (see Supplementary material online, *Table S4*). As for the HAS-BLED score, when modelled as a continuous variable, its discriminative ability was similar to what was observed in the main analyses, ranging from modest to poor (AUC from 0.55 to 0.61), across all study cohorts (see Supplementary material online, *Table S5*). Using primary care data alone to construct the HAS-BLED, reduced the discriminative ability by an average of 3% across all groups, especially for the lung cancer cohort, with an AUC of 0.54 (95% CI: 0.46–0.62), compared with an AUC of 0.59 (95% CI: 0.51–0.67) in the main analyses (see Supplementary material online, *Table S6*). In both sensitivity analyses, calibration results were similar to the main analyses, indicating very good calibration across all groups (see Supplementary material online, *Tables S5* and *S6*).

We have also performed a sensitivity analysis where cancer was defined as cancer diagnosed within 2 years before AF (see Supplementary material online, *Table S7*). The AUC values (overall) remained similar across the two definitions for cancer, and the models reported modest to good performance in discrimination for the CHA_2_DS_2_-VASc score, across all cancer cohorts. The only exception was prostate cancer, with the AUC value shifting from good to modest, with the 2-year cancer definition. In Supplementary material online, *Table S8*, the AUC values overall remained between modest to poor estimation for the HAS-BLED score across all cancer cohorts, whether cancer was diagnosed at any time before AF or 2 years before AF. There may have been a small improvement from poor to modest AUC value for the breast cancer cohort with the 2-year cancer definition, although CIs are quite wide across both AUC estimates. In addition, we have repeated the AUC estimation and included only patients who did not take OAC during the 1-year period of the study. For the CHA_2_DS_2_-VASc score (see Supplementary material online, *Table S9*), it seems that the score performs better in predicting stroke risk among patients who did not take OACs, except for the lung cancer cohort. This means that including patients who were treated with OAC has slightly affected the performance of the CHA_2_DS_2_-VASc score and made it underestimate the overall risk of stroke. Similarly, for HAS-BLED score (see Supplementary material online, *Table S10*), we observed improvements in score performance when we excluded patients treated with OACs. This implies that including OAC users has affected the performance of HAS-BLED score and made it less accurate in predicting major bleeding events.

### Net reclassification index and integrated discrimination index

During follow-up, 7124 patients developed ischaemic stroke and 134 672 patients did not develop stroke. *Table 3* describes the summary of diagnostic utilities for the original risk assessment scores (CHA_2_DS_2_-VASc and HAS-BLED) and when cancer was added as an additional predictor. For CHA_2_DS_2_-VASc score, both the original score and the one with cancer showed a sensitivity of 63.6% vs. 62% and high PPV (both 98.8%), with low specificity of 22.6% vs. 23.5% and low NPV (both 0.6%). We also calculated the changes for the CHA_2_DS_2_-VASc score, when different cancer types were added as an additional risk factor for the 7124 cases, and assumed a 10% cut-off point (probability of stroke or bleeding in case of HAS-BLED is ≥10% and assumed as high risk that equate to outcome prediction). The original CHA_2_DS_2_-VASc model misclassified 4040 stroke cases and 19 110 patients without stroke, misclassification rate 56.7% and 14.2%, respectively. When each cancer type was added as an additional factor to the CHA_2_DS_2_-VASc score, misclassification rates were higher for people with cancer; 57.5% (56 cases) for breast (NRI = −0.004; *P* < 0.001), 57.2% (39 cases) for colorectal (NRI = −0.003; *P* < 0.001), and 57.1% (30 cases) for haematological (NRI = −0.002; *P* = 0.001) (see Supplementary material online, *Table S11*). Misclassification in prostate and lung cancer cohorts did not differ significantly to the original model. As for major bleeding, 6462 patients developed bleeding and 135 334 patients did not develop bleeding during follow-up. For HAS-BLED score, both the original score and the one with cancer showed a sensitivity of 72.6% vs., 71.7% and high PPV (both 96.9%), with low specificity of 14.2% vs. 15% and low NPV (both 1.4%) (*Table 3*). The original HAS-BLED misclassified 6359 cases and 885 patients without bleeding, misclassification rate 98.4% and 0.65%, respectively. Adding breast, prostate, colorectal, and lung cancer as additional risk factors to the HAS-BLED did not result in significant changes to the NRI (see Supplementary material online, *Table S12*). Whereas, adding haematological cancer as a factor slightly improved the HAS-BLED classification of cases, misclassification rate 98.2% (NRI = 0.001, *P* = 0.043).

**Table 3:** Results of diagnostic utilities for CHA_2_DS_2_VASc and HAS-BLED, described for the original scores and when cancer is added as a covariate

Risk score	Sensitivity	Specificity	PPV	NPV	Risk estimation	Outcome event		Total
						True	False	
CHA_2_DS_2_VASc	63.6% (62–63.2)	22.6% (22.1–23.2%)	98.8% (98.6–98.9%)	0.6% (0.5–0.7%)	Negative (low risk)	11 679 (8.7%)	80 (1.1%)	11 759
					Positive (intermediate/high risk)	7044 (98.9%)	122 993 (91.3%)	130 037
CHA_2_DS_2_VASc + Cancer	62% (61–63%)	23.5% (23–24%)	98.8% (98.6–99%)	0.6% (0.5–0.7%)	Negative (low risk)	11 362 (8.4%)	78 (1.1%)	11 440
					Positive (intermediate/high risk)	7046 (98.9%)	123 310 (91.6%)	130 356
HAS-BLED	72.6% (72.2–73%)	14.2% (13.9–14.6%)	96.9% (96.7–97%)	1.4% (1.3–1.5%)	Negative (low risk)	45 682 (33.8%)	1257 (19.5%)	46 939
					Positive (intermediate/high risk)	5205 (80.5%)	89 652 (66.2%)	94 857
HAS-BLED + Cancer	71.7% (71.2–72.1%)	15% (14.6–15.3%)	96.9% (96.8–97.1%)	1.4% (1.3–1.5%)	Negative (low risk)	42 825 (31.6%)	1135 (17.6%)	43 960
					Positive (intermediate/high risk)	5327 (82.4%)	92 509 (68.4%)	97 836

PPV, positive predictive values; NPV, negative predictive values.

## Discussion

This population-based cohort study examined the performance of CHA_2_DS_2_-VASc score and HAS-BLED score in predicting ischaemic stroke and major bleeding events in patients with AF and certain types of cancer. We found that in each cancer cohort, patients tended to be older than patients without cancer, and exhibited a higher prevalence of chronic conditions. Whilst both the CHA_2_DS_2_-VASc score and HAS-BLED score showed good calibration in all study cohorts, their discriminative ability varied. The CHA_2_DS_2_-VASc score showed good discrimination in all groups but performed modestly in lung cancer cohort (C-statistic 0.69). Whereas HAS-BLED score showed modest discrimination in the cohort without cancer and poor discrimination in all cancer cohorts (C-statistic <0.6). Our analysis of a large national primary and secondary care settings highlights the potential limitations of the current stroke/bleeding risk assessment scores when used for AF patients with different types of cancer.

When the CHA_2_DS_2_-VASc score was first developed, it performed modestly in predicting ischaemic stroke (C-statistic around 0.60) in a relatively small cohort from the European Heart Survey.^20^ Later on, the score was incorporated in the 2010 ESC guidelines for the management of AF and started being used in the USA.^29^ Previous validation studies that assessed the performance of CHA_2_DS_2_-VASc score, did not specifically investigate model performance by cancer type. However, earlier studies have examined the risk of thrombo-embolic events in AF patients with cancer while using the CHA_2_DS_2_-VASc score as a risk assessment tool. In a study by Elbadawi *et al.*^30^ that looked at in-hospital cerebrovascular accidents (CVA) in AF patients with cancer have found that cancer diagnosis may not add a predictive role for in-hospital CVA beyond the CHA_2_DS_2_-VASc score. Another study investigated the risk of thrombo-embolism associated with CHA_2_DS_2_-VASc score in AF patients with and without recent cancer (diagnosed 5 years or fewer before AF) and found that the increase in CHA_2_DS_2_-VASc score was associated with a dissimilar increase in the risk of thrombo-embolism between AF patients with and without recent cancer.^31^ The investigators of that study have explained this association by the pronounced competing risk of all-cause death in patients with recent cancer. In addition, previous studies have suggested differences in the pathogenesis of stroke in cancer patients compared with patients without cancer.^32,33^

In addition, it has been previously shown that including cancer as an additional factor to the score does lead to better discriminative ability.^34^ In our analysis, both original scores (CHA_2_DS_2_-VASc and HAS-BLED) have performed similarly to the scores where cancer was additionally included, in terms of sensitivity, specificity, PPV, and NPV. We have observed a high proportion of false positive classification in both CHA_2_DS_2_-VASc score and HAS-BLED score, for both the original and modified scores. This was also reflected in the very low NPV for both scores. In the case of the CHA_2_DS_2_-VASc score, this does not necessarily translate into the score overestimating stroke risk, especially when considering that >50% of the study population have received OACs at some point during follow-up that could have reduced their risk of stroke. This is particularly relevant given that the CHA_2_DS_2_-VASc score is designed to identify low-risk patients in whom anticoagulant treatment should be avoided.^6^ We observed in our analysis that included patients who were not taking OAC and we found that CHA_2_DS_2_-VASc score allowed identification of a truly low-risk population those with CHA_2_DS_2_-VASc =0 and in whom no stroke events were recorded. However, in the case of the HAS-BLED score, this overestimation is observed despite the wide use of OACs in the cohort, which are known to increase bleeding risk. Whilst measures of predictive ability typically improve with the addition of more variables, we did not observe any improvement in the NRI when cancer was added to the CHA_2_DS_2_-VASc, but rather it led to an increase in the misclassification of patients with stroke. In addition, researchers of that study have acknowledged that their analysis was strengthened by the inclusion of 10 years of data,^34^ representing a much longer follow-up than in any previous studies of this type. Since the association between cancer and ischaemic or bleeding outcomes in patients with AF is still unclear, including cancer as an element of the CHA_2_DS_2_-VASc score, for all or some cancer types, might not constitute an improvement. This is because different cancer types may be heterogeneous in their association with stroke risk. In our analysis, we observed a proportion of 0.9–3.3% of cancer patients at low stroke risk. Based on clinical guidelines these patients are generally not offered anticoagulation. If low-risk cancer patients are unnecessarily started on OACs, this might put them at higher risk of bleeding, when the true benefit of anticoagulation in this population is still unknown. According to our findings, the CHA_2_DS_2_-VASc score exhibits good discrimination and is well calibrated in specific cancer cohorts. Based on this, the CHA_2_DS_2_-VASc seems to perform similarly in AF patients with and without, in supporting the decision to initiate anticoagulation for stroke prevention.

On the other hand, when the HAS-BLED was first developed in 2010 using data from the Euro Heart Survey, it exhibited good discriminative ability (C-statistic 0.72), for AF patients both untreated or treated with OACs.^21^ Later on, many validation studies compared its predictive accuracy with other bleeding risk assessment scores. In a meta-analysis that included 28 studies, the pooled C-statistic for the association between the HAS-BLED and major bleeding, in anticoagulated patients with AF, was 0.63, demonstrating modest predictive ability.^35^ In our analysis, we focused on assessing the performance of HAS-BLED in specific cancer cohorts. We found the HAS-BLED performed poorly in predicting major bleeding events, not only in AF patients in general but also in those with history of cancer. The analysis of clinical characteristics of patients with AF and cancer showed important differences between cancer groups in terms of bleeding risk and rate of bleeding events. This implies that cancer patients should not be considered as a homogeneous group but rather each type of cancer has different potential for serious bleeding during clinical assessment or risk prediction.^36^ This was also demonstrated in a previous study that used data from a national hospitalization database in France to compare the performance of the HAS-BLED score with other bleeding risk assessment scores in AF patients with cancer.^37^ The authors found that the C-statistic for HAS-BLED score in predicting intracranial haemorrhage ranged between 0.61 and 0.75 across 15 different types of cancer.^37^ These findings differ from what we observed, as our discrimination analysis for the HAS-BLED in different cancer types showed unsatisfactory discriminative values. This is expected when considering different AF populations and study designs; for example, we examined major bleeding events as an overall outcome, rather than specifying the type of bleeding. Nevertheless, our analysis showed that in certain cancer cohorts, the HAS-BLED score did not perform well. This is rather concerning for a score that is considered as a standard risk assessment tool in clinical practice and is recommended by clinical guidelines.^6^

Although our results have showed that the HAS-BLED score has excellent agreement between the predicted absolute risk and the true (observed) risk when patients were discriminated into different risk groups, it failed to accurately categorize patients into those at higher and lower risk of bleeding. This may in part be explained by the dynamic nature of the risk factors included in the HAS-BLED score, such as uncontrolled hypertension, labile INR, drug use, and anticoagulation. Therefore, patients who were classified as low risk at baseline may shift to the high-risk group at the end of follow-up and vice versa. This was demonstrated in a study by Chao *et al.*,^38^ which found that the prediction values of the follow-up HAS-BLED score were better compared with the baseline HAS-BLED score. Current evidence supports the HAS-BLED score because of its simple and practical application in the routine care of AF patients. However, based on our findings, relying on the HAS-BLED as a sole assessment strategy to flag up high bleeding-risk patients in the general AF population (regardless of cancer history) calls for re-evaluation.

### Strength and limitations

There are a number of strengths to this investigation. Our analysis represents the largest study to date, that enabled inclusion of a large sample of individuals representative of the population of England. The CPRD database with linkage to secondary-care database and mortality records encapsulates electronic health record data on AF patients admitted in secondary care with ischaemic stroke or major bleeding events. We applied stringent inclusion criteria to include only individuals with a first diagnosis of AF to increase the validity of the presented results. Additionally, our analysis showed how using data from primary care records only to investigate stroke or bleeding risks in AF patients could be less accurate than using linked data from primary care, secondary care, and mortality records.

Despite these strengths, there are a number of limitations inherent in observational studies. In risk prediction studies, findings are dependent on the accurate recording of risk factors. Lack of event recording would result in a false negative classification of a certain event and therefore could potentially bias findings. However, considering the clinical significance of the event, we would expect the recording for these events to be relatively complete. Another important limitation in this study was that it was not based on cancer registration data, but mainly by using a primary care database (albeit supplemented with national hospital admissions data to minimize misclassification), data on risk factors for cancer patients may not be complete, and therefore residual confounding is likely to remain. Also, our analysis has only focused on short-term risk prediction (stroke or bleeding events within 1 year from AF diagnosis), therefore our findings cannot be generalized to patients with chronic AF (AF diagnosed > 1 year). Finally, we decided to use a modified HAS-BLED score of eight points rather than the complete score with nine points due to lack of consistency in INR recording in CPRD. Labile INR as a risk factor in HAS-BLED could have influenced the predictive performance of the model either to over- or underestimate the rate of bleeding.

## Conclusion

We found that amongst people with AF and certain types of cancer, the CHA_2_DS_2_-VASc score performs similarly in predicting ischaemic stroke in patients with AF without cancer, and has good discrimination and calibration in cancer sub-groups. Whereas the HAS-BLED score despite being well calibrated does not have good discrimination in the AF population in general and in specific cancer cohorts when predicting major bleeding events. Therefore, it seems that the performance of the HAS-BLED in the cancer population is suboptimal, which may lead to over- or underestimation of bleeding risk, hence, it will influence the decision to prescribe anticoagulants. Our findings highlight the importance of cancer diagnosis during the development of risk scores and highlight opportunities to optimize the HAS-BLED risk score to better serve cancer patients with AF.

## Supplementary Material

oeae053_Supplementary_Data

## Data Availability

This study was conducted using CPRD data obtained under licence from the UK Medicines and Healthcare Products Regulatory Agency. The data were requested via application to CPRD and approved by the CPRD's Independent Scientific Advisory Committee (ISAC) (protocol number: 20_198R). The raw data underlining the results presented in the study are subject to CPRD’s Research Data Governance process (https://cprd.com/Data-access, contact enquiries@cprd.com). The data are provided by patients and collected by the NHS as part of their care and support. The Office for National Statistics is the provider of the ONS data contained within the CPRD data. Hospital Episode Data and the ONS data, © 2021, are reused with the permission of NHS Digital. All rights reserved. We are grateful to the contributing patients and practices in the CPRD who have allowed their data to be used for research purposes.
