# Supplementary material for: Performance of CHA2DS2-VASc and HAS-BLED in predicting stroke and bleeding in atrial fibrillation and cancer
Source: Eur Heart J Open. 2024 Jun 26;4(4):oeae053. doi: 10.1093/ehjopen/oeae053 (PMC11234295; doi:10.1093/ehjopen/oeae053)
Supplement: oeae053_Supplementary_Data [file oeae053_supplementary_data.docx]

**Figure S1: Kaplan-Meier curve of cumulative stroke events over one year observation period.**

**
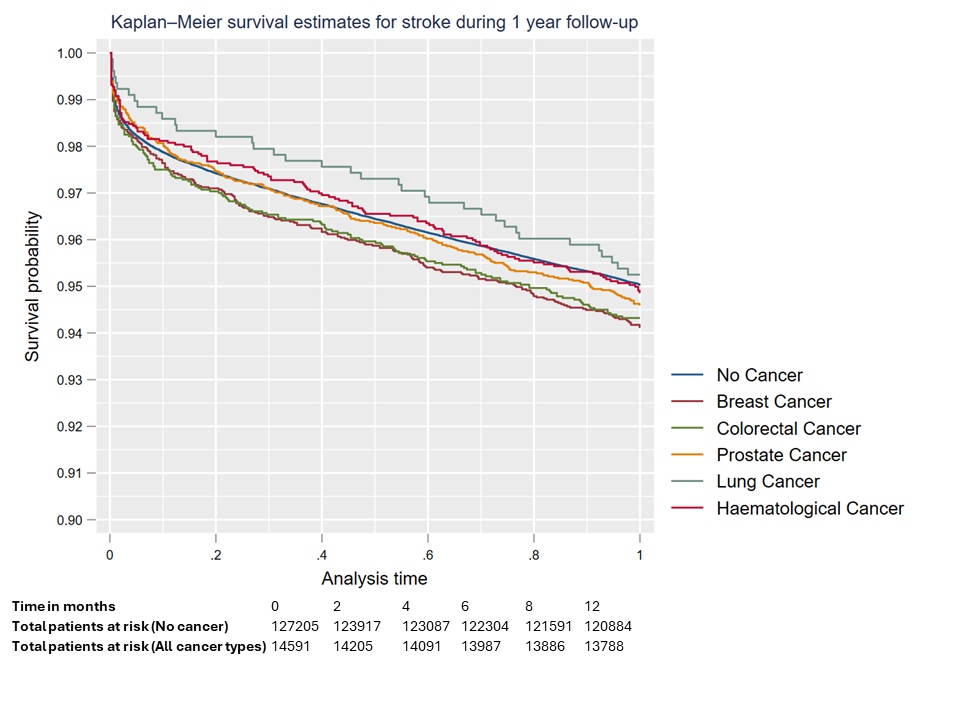
**

**Figure S2: Kaplan-Meier curve of cumulative bleeding events over one year observation period.**


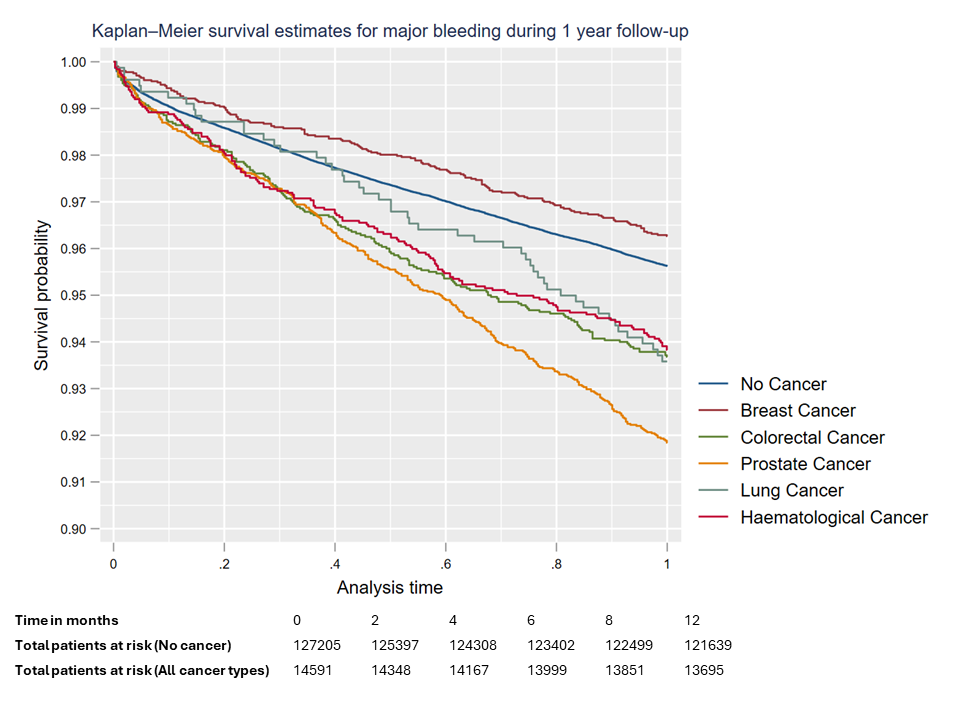


**Table S1: Clinical characteristics composing the CHA_2_DS_2_VASc and HAS-BLED scores.**

| **CHA_2_DS_2_VASc** | | **HAS-BLED** | |
| --- | --- | --- | --- |
| **Clinical characteristics** | **Points** | **Clinical characteristics** | **Points** |
| Congestive heart failure | 1 point | Uncontrolled hypertension (SBP) >160 mm Hg | 1 point |
| Hypertension | 1 point | Abnormal kidney function/ liver function | 1 point each |
| Age 65 to 74 years | 1 point | Previous stroke | 1 point |
| Age ≥75 years | 2 points | Bleeding history or predisposition | 1 point |
| Diabetes | 1 point | Labile INR | NA |
| Stroke | 2 points | Elderly (age >65 years) | 1 point |
| Sex category (female) | 1 point | Drug use (antiplatelet, nonsteroidal anti‐inflammatory drugs)/alcohol abuse) | 1 point each |
| Total | 9 points | Total | 8 points |

Abbreviations: INR, International Normalised Ratio; SBP, Systolic Blood Pressure.

**Table S2: Stroke events at 1-year based on CHA_2_-DS_2_-VASc score value in patients with AF (with and without cancer) who did not receive anticoagulation therapy.**

| CHA_2_-DS_2_-VASc score | AF cohort (no cancer) | | AF and cancer cohort | |
| --- | --- | --- | --- | --- |
|  | Number of events | Event rates and 95% CI | Number of events | Event rates and 95% CI |
| 0 | 7 | 0.2% (0.1%; 0.3%) | 0 | 0% |
| 1 | 25 | 0.5% (0.3%; 0.7%) | 1 | 0.3% (0.1%; 2.3%) |
| 2 | 62 | 1.3% (1.0%; 1.6%) | 4 | 0.7% (0.3%; 1.9%) |
| 3 | 98 | 2.0% (1.6%; 2.4%) | 12 | 1.6% (0.9%; 2.9%) |
| 4 | 194 | 3.8% (3.3%; 4.4%) | 18 | 2.5% (1.6%; 4.0%) |
| 5 | 208 | 6.4% (5.6%; 7.3%) | 28 | 5.7% (4.0%; 8.2%) |
| 6 | 204 | 10.2% (9.0%; 11.6%) | 24 | 8.5% (5.8%; 12.4%) |
| 7 | 152 | 14.9% (12.8%; 17.2%) | 27 | 18.0% (12.6%; 25.0%) |
| 8 | 56 | 15.5% (12.1%; 19.6%) | 10 | 20.4% (11.4%; 33.9%) |
| 9 | 22 | 31.0% (21.4%; 42.6%) | 4 | 28.6% (11.1%; 56.1%) |

**Table S3: Sensitivity analysis for CHA_2_-DS_2_-VASc discrimination (modelled as continuous variable)**

| **Discrimination** | | | | |
| --- | --- | --- | --- | --- |
| **Cancer status** | **C-statistics** | | **95% CI** | |
| **No cancer** | 0.732 | | 0.726 to 0.738 | |
| **Breast cancer** | 0.688 | | 0.654 to 0.723 | |
| **Prostate cancer** | 0.741 | | 0.710 to 0.772 | |
| **Colorectal cancer** | 0.704 | | 0.661 to 0.747 | |
| **Haematological cancer** | 0.714 | | 0.669 to 0.760 | |
| **Lung Cancer** | 0.693 | | 0.601 to 0.785 | |
| **Calibration** | | | | |
| **Cancer status** | **E:O** | **CITL** | | **Slope** |
| **No cancer** | 1.000 | 0.000 | | 1.000 |
| **Breast cancer** | 1.000 | -0.000 | | 1.000 |
| **Prostate cancer** | 1.000 | 0.000 | | 1.000 |
| **Colorectal cancer** | 1.000 | -0.000 | | 1.000 |
| **Haematological cancer** | 1.000 | 0.000 | | 1.000 |
| **Lung Cancer** | 1.000 | -0.000 | | 1.000 |

**Table S4: Sensitivity analysis for CHA_2_-DS_2_-VASc discrimination (score components assessed using CPRD only)**

| **Discrimination** | | | | |
| --- | --- | --- | --- | --- |
| **Cancer status** | **C-statistics** | | **95% CI** | |
| **No cancer** | 0.678 | | 0.672 to 0.685 | |
| **Breast cancer** | 0.647 | | 0.610 to 0.684 | |
| **Prostate cancer** | 0.705 | | 0.671 to 0.739 | |
| **Colorectal cancer** | 0.662 | | 0.616 to 0.707 | |
| **Haematological cancer** | 0.687 | | 0.638 to 0.735 | |
| **Lung Cancer** | 0.606 | | 0.507 to 0.705 | |
| **Calibration** | | | | |
| **Cancer status** | **E:O** | **CITL** | | **Slope** |
| **No cancer** | 1.000 | -0.000 | | 1.000 |
| **Breast cancer** | 1.000 | -0.000 | | 1.000 |
| **Prostate cancer** | 1.000 | 0.000 | | 1.000 |
| **Colorectal cancer** | 1.000 | -0.000 | | 1.000 |
| **Haematological cancer** | 1.000 | -0.000 | | 1.000 |
| **Lung Cancer** | 1.000 | -0.000 | | 1.000 |

**Table S5: Sensitivity analysis for HAS-BLED discrimination/calibration (modelled as continuous variable)**

| **Discrimination** | | | | |
| --- | --- | --- | --- | --- |
| **Cancer status** | **C-statistics** | | **95% CI** | |
| **No cancer** | 0.615 | | 0.607 to 0.622 | |
| **Breast cancer** | 0.556 | | 0.510 to 0.603 | |
| **Prostate cancer** | 0.577 | | 0.547 to 0.607 | |
| **Colorectal cancer** | 0.569 | | 0.525 to 0.612 | |
| **Haematological cancer** | 0.592 | | 0.547 to 0.638 | |
| **Lung Cancer** | 0.553 | | 0.472 to 0.634 | |
| **Calibration** | | | | |
| **Cancer status** | **E:O** | **CITL** | | **Slope** |
| **No cancer** | 1.000 | -0.000 | | 1.000 |
| **Breast cancer** | 1.000 | 0.000 | | 1.000 |
| **Prostate cancer** | 1.000 | -0.000 | | 1.000 |
| **Colorectal cancer** | 1.000 | -0.000 | | 1.000 |
| **Haematological cancer** | 1.000 | -0.000 | | 1.000 |
| **Lung Cancer** | 1.000 | -0.000 | | 1.000 |

**Table S6: Sensitivity analysis for HAS-BLED discrimination/calibration (score components assessed using CPRD only)**

| **Discrimination** | | | | |
| --- | --- | --- | --- | --- |
| **Cancer status** | **C-statistics** | | **95% CI** | |
| **No cancer** | 0.588 | | 0.581 to 0.596 | |
| **Breast cancer** | 0.564 | | 0.517 to 0.611 | |
| **Prostate cancer** | 0.560 | | 0.528 to 0.592 | |
| **Colorectal cancer** | 0.546 | | 0.502 to 0.590 | |
| **Haematological cancer** | 0.563 | | 0.514 to 0.611 | |
| **Lung Cancer** | 0.545 | | 0.464 to 0.625 | |
| **Calibration** | | | | |
| **Cancer status** | **E:O** | **CITL** | | **Slope** |
| **No cancer** | 1.000 | -0.000 | | 1.000 |
| **Breast cancer** | 1.000 | 0.000 | | 1.000 |
| **Prostate cancer** | 1.000 | -0.000 | | 1.000 |
| **Colorectal cancer** | 1.000 | -0.000 | | 1.000 |
| **Haematological cancer** | 1.000 | 0.000 | | 1.000 |
| **Lung Cancer** | 1.000 | -0.000 | | 1.000 |

**Table S7: AUC and 95% CI for** **CHA_2_DS_2_-VASc based on cancer type**

| **Cancer status** | **AF**  **(no cancer)** | **Breast cancer** | **Prostate cancer** | **Colorectal cancer** | **Haematological cancer** | **Lung cancer** |
| --- | --- | --- | --- | --- | --- | --- |
| **Any time before AF** | 0.73  (0.72; 0.74) | 0.70  (0.66; 0.74) | 0.74  (0.71; 0.77) | 0.70  (0.66; 0.75) | 0.71  (0.66; 0.76) | 0.69  (0.60; 0.79) |
| **Two years before AF** | 0.72  (0.71; 0.73) | 0.72  (0.71; 0.78) | 0.69  (0.67; 0.70) | 0.70  (0.67; 0.73) | 0.69  (0.66; 0.71) | 0.70  (0.66; 0.74) |

**Table S8: AUC and 95% CI for HAS-BLED based on cancer type**

| **Cancer status** | **AF**  **(no cancer)** | **Breast cancer** | **Prostate cancer** | **Colorectal cancer** | **Haematological cancer** | **Lung cancer** |
| --- | --- | --- | --- | --- | --- | --- |
| **Any time before AF** | 0.61  (0.60: 0.62) | 0.56  (0.52; 0.61) | 0.58  (0.55; 0.61) | 0.57  (0.53; 0.61) | 0.59  (0.55; 0.64) | 0.59  (0.51; 0.67) |
| **Two years before AF** | 0.61  (0.60; 0.62) | 0.61  (0.59; 0.64) | 0.56  (0.54; 0.58) | 0.56  (0.53; 0.59) | 0.59  (0.57; 0.62) | 0.62  (0.58; 0.66) |

**Table S9: comparing AUC and 95% CI for CHA_2_DS_2_VASc from the main analysis vs.** **when excluding OAC users**

| **Cancer status** | **AF**  **(no cancer)** | **Breast cancer** | **Prostate cancer** | **Colorectal cancer** | **Haematological cancer** | **Lung cancer** |
| --- | --- | --- | --- | --- | --- | --- |
| **Main analysis**  **(OAC users and non-users)** | 0.73  (0.72; 0.74) | 0.70  (0.66; 0.74) | 0.74  (0.71; 0.77) | 0.70  (0.66; 0.75) | 0.71  (0.66; 0.76) | 0.69  (0.60; 0.79) |
| **Excluding OAC users** | 0.79  (0.78; 0.80) | 0.71  (0.68; 0.74) | 0.74  (0.70; 0.78) | 0.78  (0.74; 0.82) | 0.74  (0.70; 0.78) | 0.65  (0.56; 0.74) |

**Table S10: comparing AUC and 95% CI for HAS-BLED from the main analysis vs. when excluding OAC users**

| **Cancer status** | **AF**  **(no cancer)** | **Breast cancer** | **Prostate cancer** | **Colorectal cancer** | **Haematological cancer** | **Lung cancer** |
| --- | --- | --- | --- | --- | --- | --- |
| **Main analysis**  **(OAC users and non-users)** | 0.61  (0.60: 0.62) | 0.56  (0.52; 0.61) | 0.58  (0.55; 0.61) | 0.57  (0.53; 0.61) | 0.59  (0.55; 0.64) | 0.59  (0.51; 0.67) |
| **Excluding OAC users** | 0.67  (0.66; 0.68) | 0.69  (0.66; 0.72) | 0.65  (0.61; 0.68) | 0.58  (0.54; 0.62) | 0.66  (0.62; 0.70) | 0.61  (0.54; 0.67) |

**Table S11: Risk classification and NRI estimates of CHA_2_DS_2_VASc score when adding cancer type as a risk factor**

|  | | **Cancer cohort** | | | | | | | | | |
| --- | --- | --- | --- | --- | --- | --- | --- | --- | --- | --- | --- |
|  |  | **Breast cancer** | | **Prostate cancer** | | **Colorectal cancer** | | **Haematological cancer** | | **Lung cancer** | |
| **NRI estimate (95% CI and P value)** | | -0.004  P< 0.001 | | 0.000  P < 0.001 | | -0.003  P< 0.001 | | -0.002  P=0.001 | | -0.000  P= 0.334 | |
| **IDI estimate (95% CI and P value)** | | 0.000  P= 0.033 | | 0.000  P< 0.001 | | 0.000  P< 0.001 | | 0.000  P= 0.329 | | 0.000  P= 0.567 | |
| **Outcome during 1-year follow up** | **Risk classification in original CHA_2_DS_2_VASc** | **Risk classification in CHA_2_DS_2_VASc + cancer as an additional risk factor** | | | | | | | | | |
|  |  | **<10%** | **≥10%** | **<10%** | **≥10%** | **<10%** | **≥10%** | **<10%** | **≥10%** | **<10%** | **≥10%** |
| **Stroke** | **<10%** | 4040 | 0 | 4040 | 0 | 4040 | 0 | 4040 | 0 | 4040 | 0 |
|  | **≥10%** | 56 | 3028 | 0 | 4084 | 39 | 3045 | 30 | 3054 | 6 | 3078 |
| **No stroke** | **<10%** | 115,562 | 0 | 115,562 | 0 | 115,562 | 0 | 115,562 | 0 | 115,562 | 0 |
|  | **≥10%** | 463 | 18,647 | 0 | 19,110 | 309 | 18,801 | 241 | 18,869 | 68 | 19,042 |

**Table S12: Risk classification and NRI estimates of HAS-BLED score when adding cancer type as a risk factor**

|  | | **Cancer cohort** | | | | | | | | | |
| --- | --- | --- | --- | --- | --- | --- | --- | --- | --- | --- | --- |
|  |  | **Breast cancer** | | **Prostate cancer** | | **Colorectal cancer** | | **Haematological cancer** | | **Lung cancer** | |
| **NRI estimate (95% CI and P value)** | | 0.000  P= 0.923 | | 0.009  P <0.001 | | 0.000  P= 0.195 | | 0.001  P= 0.043 | | 0.000  P= 0.401 | |
| **IDI estimate (95% CI and P value)** | | 0.000  P< 0.001 | | 0.000  P <0.001 | | 0.000  P= 0.028 | | 0.000  P= 0.018 | | 0.000  P= 0.124 | |
| **Outcome during 1-year follow up** | **Risk classification in original HAS-BLED** | **Risk classification in HAS-BLED + cancer as an additional risk factor** | | | | | | | | | |
|  |  | **<10%** | **≥10%** | **<10%** | **≥10%** | **<10%** | **≥10%** | **<10%** | **≥10%** | **<10%** | **≥10%** |
| **Bleeding** | **<10%** | 6359 | 0 | 6254 | 105 | 6347 | 12 | 6346 | 13 | 6356 | 3 |
|  | **≥10%** | 1 | 102 | 0 | 103 | 0 | 103 | 0 | 103 | 0 | 103 |
| **No bleeding** | **<10%** | 134,449 | 0 | 133,586 | 863 | 134,293 | 156 | 134,331 | 118 | 134,417 | 32 |
|  | **≥10%** | 23 | 862 | 0 | 885 | 0 | 885 | 0 | 885 | 0 | 885 |
